# Associations of magnesium depletion score with the incidence and mortality of osteoarthritis: a nationwide study

**DOI:** 10.3389/fimmu.2025.1512293

**Published:** 2025-02-28

**Authors:** Ruicong Ma, Cheng Zhang, Jiaqing Liu, Jinyi Ren, Huina Huang, Guan Wang, Yanchun Ding, Xia Li

**Affiliations:** ^1^ Department of Cardiology, The Second Hospital of Dalian Medical University, Dalian, Liaoning, China; ^2^ Department of Immunology, College of Basic Medical Science, Dalian Medical University, Dalian, Liaoning, China

**Keywords:** osteoarthritis, NHANES, magnesium depletion score, all-cause mortality, cardiovascular mortality

## Abstract

**Background:**

Magnesium is an essential immune nutrient for the body, and recent studies have found that it plays an important role in osteoarthritis (OA). Magnesium depletion score(MDS) is a new method for evaluating the magnesium status of the body. Our objective is to explore the association between MDS and the incidence of OA, as well as the relationship between MDS and mortality in patients with OA.

**Methods:**

Eligible participants were obtained from NHANES from 2005 to 2018. Logistic regression models were employed to evaluate the link between MDS and the incidence of OA. Cox regression models were employed to evaluate the link between MDS and mortality among OA patients. In addition, restricted cubic spline was utilized to explore the correlation between MDS and the incidence of OA, as well as the relationship between MDS and mortality in patients with OA. Subgroup analysis were adopted in order to ensure the credibility of the results in different subgroups, including age, gender, race, education level, BMI, smoking, diabetes and hypertension.

**Results:**

19,394 individuals qualified for analysis, including 3,256 OA patients. After excluding missing follow-up data, 630 all-cause deaths and 172 cardiovascular deaths (CVDs) were observed in 3,250 OA patients. The individuals with OA had higher levels of MDS. In the logistic regression model, MDS was positively related to OA (MDS≥3 vs. MDS=0, OR =1.83 (1.46-2.30, *P*<0.001)). Besides, a positive association was observed between MDS and all-cause mortality [MDS≥3 vs. MDS=0, HR =2.56 (1.49-4.41, *P*<0.001)] and CVDs [MDS≥3 vs. MDS=0, HR =3.00 (1.13-7.98, *P*=0.01)] in cox regression models. In addition, a 1-unit rise in MDS was significantly linked to an increased risk of mortality. Restricted cubic spline indicated a positive relationship between MDS and incidence and mortality of OA. Subgroup analysis demonstrated that the results are stable in different subgroups.

**Conclusions:**

MDS is positively correlated with the incidence and mortality of OA. Optimizing the nutritional status of magnesium may bring benefits to OA patients.

## Introduction

Osteoarthritis(OA) is a degenerative condition of the joints marked by stiffness, pain, and deformities (1). OA is particularly prevalent among middle-aged and elderly populations, affecting approximately 595 million people worldwide (2). OA affects about 14% of American adults. In addition, it is the second leading cause of labor loss, second only to cardiovascular disease. OA causes medical expenses and other economic losses of up to about $125 billion annually (3). Patients with OA might face an increased risk of mortality in comparison to the overall population (4–6). Although the prevention and surgical treatment measures have been effectively implemented, the incidence and mortality of OA are still difficult to control.

Recently, some studies have found that mineral elements are crucial in the development of OA (7, 8). Magnesium is one of the most important and abundant trace elements in cells. It is an auxiliary factor in enzymatic reactions (9). Furthermore, many metabolic reactions in the human body, including the production of ATP and the maintenance of normal mitochondrial function, rely on the participation of magnesium (10). Magnesium supplementation can reduce joint cartilage damage, apoptosis, and promote chondrocyte generation (11, 12). Moreover, administering intra-articular magnesium injections can help reduce pain in both OA rats and patients (13, 14). Therefore, magnesium plays an important role in OA.The National Institutes of Health (NIH) in the United States pointed out that assessing magnesium levels in the body is very difficult due to the fact that most magnesium remains in tissues or cells. The detection of serum magnesium in clinical practice is not accurate (15). Due to the lack of an effective method for evaluating the magnesium status of the body at present, the significance of magnesium deficiency in OA has been largely neglected.

The magnesium depletion score (MDS) serves as an effective instrument for evaluating the body’s magnesium levels. An increase in MDS indicates severe magnesium deficiency in the individual. Fan et al. found that MDS has greater value in evaluating the magnesium status of the body compared with serum magnesium. Furthermore, high MDS may indicate an inflammatory state which is linked to increased long-term mortality in individuals (16). In addition, other studies also revealed that an elevation in MDS is related to an increased risk of abdominal aortic calcification, cardiovascular disease and diabetes retinopathy (17–19). Nonetheless, the relationship between MDS and the incidence and mortality of OA remains ambiguous. Therefore, our objective is to explore the association between MDS and the incidence of OA, as well as the relationship between MDS and mortality in patients with OA.

## Materials and methods

### Data source and study population

Data for this research were sourced from the National Health and Nutrition Examination Survey (NHANES) database (www.cdc.gov/nchs/nhanes.com).

Part I: The inclusion criteria are as follows: The participants from NHANES between 2005 and 2018. The exclusion criteria are as follows: participants younger than 40 years, participants without OA information and those missing data on MDS.

Part II: Based on Part I, we have developed inclusion and exclusion criteria. The inclusion criteria are as follows: participants with OA. The exclusion criteria are as follows: those missing data on follow-up information.

### The assessment of MDS

The MDS calculation comprised the consolidation of four separate scores: (1) the use of diuretics at present received 1 point; (2) using a proton pump inhibitor (PPI) also earned 1 point; (3) an estimated glomerular filtration rate (eGFR) ranging from 60 mL/min/1.73 m² to less than 90 mL/min/1.73 m² was assigned 1 point, whereas an eGFR below 60 mL/min/1.73 m² received 2 points; (4) heavy alcohol consumption (defined as more than 1 drink per day for women and over 2 drinks per day for men) was allocated 1 point (18).

### Covariates

Demographic information encompasses age, gender, ethnicity, education levels, body mass index (BMI) and poverty income ratio (≤1.30, 1.31–3.49 and ≥3.50). Physical activity data was obtained through a questionnaire survey. Laboratory tests include total cholesterol (TC) (mmol/L), glycated hemoglobin (HbA1c) (%), calcium (mmol/L) and phosphorus (mmol/L). Dietary factors (magnesium intake, calcium intake, phosphorus intake, vitamin D intake), smoking status and comorbidities (hypertension and diabetes) are also included. Dietary data is sourced from a dietary recall survey. Oxidative stress is closely related to the occurrence and development of OA. Antioxidant diet may be an easily accepted treatment strategy. Based on previously published articles on OA, we also calculated the composite dietary antioxidant index (CDAI). It comprises a composite score of six dietary antioxidants: vitamins A, C, and E, as well as selenium, zinc, and carotenoids (20). The criteria for diagnosing hypertension include: SBP ≥ 140 mmHg or DBP ≥ 90 mmHg and the patients using antihypertensive medications (21). The criteria used for diagnosing diabetes include: doctor diagnosis as diabetes, HbA1c ≥ 6.5%, fasting glucose ≥ 7.0mmol/L, random blood glucose ≥ 11.1mmol/L, 2h OGTT blood glucose ≥ 11.1mmol/L, or the administration of diabetes medications and insulin therapy (22).

### Mortality

The mortality statistics were connected up to December 31, 2019 (https://www.cdc.gov/nchs/data-linkage/mortality.htm). Outcomes were divided into categories of all-cause deaths and CVDs. Death causes were classified according to ICD-10 codes, with CVDs specifically identified using codes 100-109, 111, 113, and 120-151 (23).

### Statistical analysis

NHANES is conducted in a complex multi-stage sampling design. Moreover, NHANES is conducted in two-year cycles and includes a representative sample of the U.S. population. Standard error of mean (SEM) reflects the representativeness of sample mean to population mean. Weighted percentages can better represent the overall population. Therefore, SEM and weighted percentage might be more appropriate. Initially, continuous variables were represented as means corresponding standard error of the mean (mean ± SEM) and categorical variables were presented as means [95% confidence intervals (CI)]. The differences between the two groups were compared using chi-square tests and independent-sample t tests. *P* values < 0.05 were recognized statistically significant. The purpose of regression analysis is to observe the degree of correlation between the dependent variable and the independent variable after adjusting for various confounding factors. To examine the relationship between MDS and the incidence of OA, weighted logistic regression analyses were conducted. Three models were developed: unadjusted, Model I, and Model II. Model I was adjusted for age, sex, and race. Model II was adjusted for age, sex, ethnicity, education levels, BMI, smoking, HbA1c, TC, hypertension, DM, calcium, phosphorus, phosphorus intake, calcium intake, magnesium intake, CDAI, vitamin D intake, physical activity and poverty income ratio. The restricted cubic spline(RCS) curve can more vividly observe the relationship between the dependent variable and the independent variable. RCS analysis was employed to investigate the link between MDS and the incidence of OA. The purpose of subgroup analysis is to divide the study population into different groups based on certain characteristics (such as age, gender, comorbidities, etc.) to observe whether experimental variables have different effects in these different groups. Additionally, subgroup analysis was conducted to determine if the relationship between MDS and the incidence of OA remained consistent across various groups. These subgroups include age, gender, race, education level, BMI, smoking, diabetes and hypertension.

Additionally, weighted Kaplan-Meier curves and log-rank tests were utilized to examine the cumulative survival differences among different MDS groups. Cox regression analysis was carried out to investigate the link between MDS and mortality with OA. The variables included in the model are consistent with cox regression analysis model. Similarly, RCS was employed to investigate the relationship between MDS and mortality. Additionally, subgroup analysis was performed to further verify the robustness of the findings.

Besides, sensitivity analysis was also used to further validate our results. In many studies, weighted and unweighted results may be inconsistent. Consequently, we conducted unweighted Cox regression to carry out sensitivity analysis. Additionally, we also excluded OA patients who died within two years to further analyze the link between MDS and mortality.

## Results

### The link between MDS and OA

**Figure 1:**
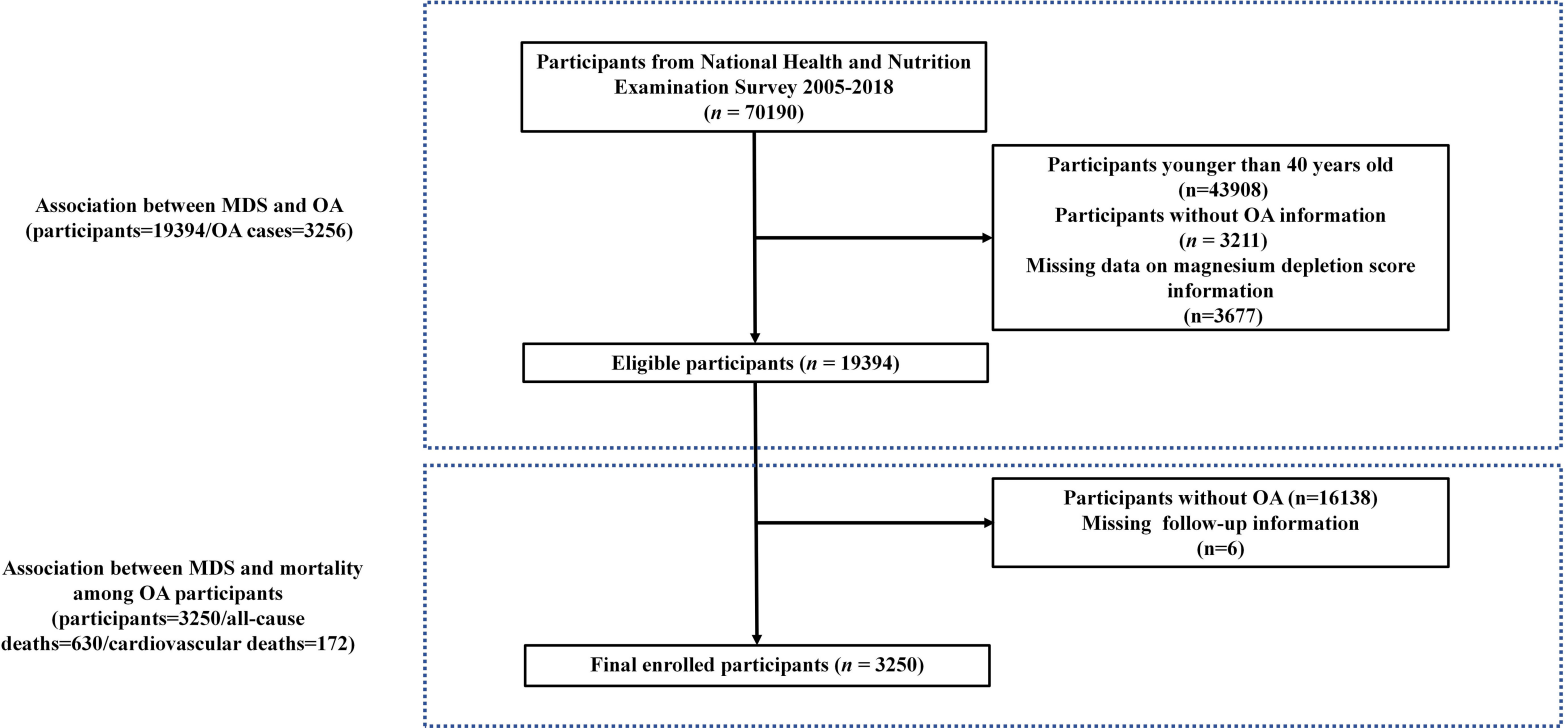
The flow chart of participant selection.

The participants from NHANES between 2005 and 2018 (n = 70,190), we eliminated subjects younger than 40 years (n = 43,908), subjects without OA information (n = 3,211), those missing data on magnesium depletion score (n = 3,677). Consequently, the cross-sectional analysis sample comprised 19,394 participants. Detailed information of the screening process is shown in **Figure 1**. 19,394 adults were screened for this cross-sectional study, including 3,256 OA patients. The clinical baseline characteristics of non-OA and OA participants are presented in **Table 1**, including age, gender, ethnicity, educational levels, BMI, HbA1c, TC, calcium, phosphorus, phosphorus intake, calcium intake and magnesium intake, smoking, hypertension, diabetes. Individuals in the OA group tend to be older (63.57 ± 0.26vs.55.72 ± 0.16), with a higher likelihood of being female (64.85%vs.49.09%), white(84.05% vs.70.77%), and former smokers (51.32%vs.46.28%), as well as a greater proportion of those who have hypertension (64.06%vs.45.73%), and diabetes (23.56%vs.18.01%)(*P*<0.001). We also found differences between laboratory examination measurements (HbA1c, TC, serum calcium and phosphorus) and dietary factors (phosphorus intake, calcium intake and magnesium intake) (*P* < 0.001). **Supplementary Table S1** presents the baseline characteristics of the participants categorized by their MDS levels. Compared with the MDS=0 group, patients in the MDS≥3 group were older (68.65 ± 0.35vs.50.61 ± 0.17), had higher BMI (30.77 ± 0.22vs.29.25 ± 0.12), HbA1c (6.01 ± 0.03vs.5.77 ± 0.02), and had a larger proportion of smoking (51.09%vs.44.55%), hypertension (86.64%vs.33.39%) and diabetes (34.11%vs.16.04%). We can also find that the incidence of OA gradually increases with the increase of MDS. In addition, patients in the OA group have a higher level of MDS (1.47 ± 0.02vs.1.01 ± 0.01). We found that OA patients had higher use of diuretics (25.56%vs.14.14%) and PPIs (21.82%vs.9.42%), as well as lower eGFR (**Table 2**), which are components of MDS.

**Table 1:** Clinical characteristics of study population.

Variables	Overall	Non-OA	OA	*P* value
Age, %	57.22±0.16	55.72±0.16	63.57±0.26	<0.001
Sex, %				<0.001
Female	52.09(49.33,54.84)	49.09(48.24,49.94)	64.85(62.94,66.76)	
Male	47.91(45.24,50.58)	50.91(50.06,51.76)	35.15(33.24,37.06)	
Race/ethnicity, %				<0.001
White	73.30(67.44,79.15)	70.77(68.23,73.31)	84.05(82.10,86.01)	
Black	9.46( 8.46,10.45)	10.25(8.90,11.60)	6.10(4.99, 7.22)	
Mexican	6.32( 5.34, 7.30)	7.21(5.99,8.42)	2.56(1.88,3.24)	
Others	10.92( 9.95,11.89)	11.78(10.58,12.98)	7.29( 6.14, 8.43)	
Education levels, %				<0.001
Less than high school	15.36(14.15,16.57)	16.14(14.94,17.34)	12.09(10.58,13.60)	
High school or equivalent	23.53(21.75,25.31)	23.61(22.43,24.80)	23.21(21.04,25.38)	
College or above	61.07(57.35,64.78)	60.24(58.40,62.09)	64.70(62.21,67.19)	
BMI, kg/m2	29.30±0.08	28.95±0.09	30.77±0.20	<0.001
HbA1c, %	5.78±0.01	5.77±0.01	5.84±0.02	<0.001
TC, mmol/L	5.17±0.01	5.19±0.01	5.13±0.02	0.02
Serum calcium, mmol/L	2.35±0.00	2.35±0.00	2.36±0.00	0.01
Serum phosphorus, mmol/L	1.20±0.00	1.19±0.00	1.22±0.00	<0.001
Magnesium intake, mg	305.46±1.92	308.01±2.17	294.60±3.46	0.001
Calcium intake, mg	940.36±7.09	946.58± 7.56	913.88±14.47	0.03
Phosphorus intake, mg	1360.89±7.11	1375.73± 7.83	1297.79±15.98	<0.001
Vitmain D intake,mcg	4.67±0.07	4.70±0.08	4.55±0.13	0.28
CDAI	0.75(0.62,0.87)	0.77(0.63,0.91)	0.65(0.45,0.85)	0.33
Physical activity, %				0.08
No	53.00(50.22,55.78)	52.55(50.92,54.18)	54.90(52.44,57.36)	
Yes	47.00(43.81,50.19)	47.45(45.82,49.08)	45.10(42.64,47.56)	
Poverty income ratio, %				0.19
≤1.30	15.50(14.27,16.72)	16.89(15.51,18.27)	15.40(13.69,17.10)	
1.31–3.49	32.27(30.15,34.39)	34.25(32.70,35.81)	35.96(33.56,38.35)	
≥3.50	45.55(42.00,49.10)	48.86(46.61,51.10)	48.65(45.43,51.87)	
Smoking, %				<0.001
No	52.74(50.02,55.46)	53.72(52.45,54.99)	48.68(46.05,51.31)	
Yes	47.22(44.19,50.26)	46.28(45.01,47.55)	51.32(48.69,53.95)	
Hypertension, %				<0.001
No	50.78(47.70,53.85)	54.27(52.93,55.62)	35.94(33.52,38.36)	
Yes	49.22(46.55,51.88)	45.73(44.38,47.07)	64.06(61.64,66.48)	
DM, %				<0.001
No	80.86(76.34,85.39)	81.99(81.10,82.88)	76.44(74.54,78.33)	
Yes	19.05(17.92,20.19)	18.01(17.12,18.90)	23.56(21.67,25.46)	

Continuous data were presented as the mean±SEM, category data were presented as the proportion and 95% confidence interval. SEM, Standard Error of the Mean; BMI, body mass index; HbA1c, glycosylated hemoglobin; MDS, Magnesium depletion score; TC, total cholesterol; CDAI, composite dietary antioxidant index; DM, diabetes mellitus.

**Table 2:** MDS and its components among non-OA group and OA group.

Variables	Overall	Non-OA	OA	*P* value
MDS	1.09±0.01	1.01±0.01	1.47±0.02	<0.001
Diuretic use				<0.001
No	83.69(79.12,88.25)	85.86(85.04,86.69)	74.44(72.35,76.52)	
Yes	16.31(15.21,17.41)	14.14(13.31,14.96)	25.56(23.48,27.65)	
PPI use				<0.001
No	88.22(83.70,92.74)	90.58(89.88,91.29)	78.18(76.49,79.88)	
Yes	11.78(10.74,12.82)	9.42( 8.71,10.12)	21.82(20.12,23.51)	
Heavy drinking				0.01
No	83.77(79.25,88.28)	83.26(82.16,84.37)	85.89(84.17,87.61)	
Yes	16.23(14.93,17.54)	16.74(15.63,17.84)	14.11(12.39,15.83)	
EGFR scores				<0.001
0	45.15(42.83,47.47)	48.44(47.02,49.86)	31.17(29.13,33.21)	
1	44.75(41.82,47.69)	42.91(41.66,44.15)	52.61(50.45,54.77)	
2	10.10( 9.32,10.87)	8.66( 8.07, 9.25)	16.22(14.73,17.72)	

Data were presented as the mean±SEM. SEM, Standard Error of the Mean; MDS, Magnesium depletion score; OA,osteoarthritis; PPI, proton pump inhibitor; eGFR, estimated glomerular filtration rate.

Additionally, a positive correlation was presented between MDS and the incidence of OA with an OR of 1.22 (95%CI: 1.14-1.30) in logistic regression analysis (**Table 3**). Compared with the group with MDS=0, the group with MDS≥3 has a higher incidence of OA (OR = 1.83, 95%CI: 1.46-2.30) in Model II. The use of unweighted multiple analysis as a sensitivity analysis also confirmed this result (**Supplementary Table S3**).

**Table 3:** Weighted logistic regression analysis on the association between MDS and OA.

	Non-adjusted model		Model I		Model II	
	OR [95% CI]	*P* value	OR [95% CI]	*P* value	OR [95% CI]	*P* value
Continuous MDS	1.59(1.52,1.66)	<0.001	1.25(1.19,1.32)	<0.001	1.22(1.14,1.30)	<0.001
MDS=0	Reference	–	Reference	–	Reference	–
MDS=1	1.76(1.52,2.04)	<0.001	1.28(1.08,1.50)	0.004	1.19(0.98,1.43)	0.07
MDS=2	2.79(2.39,3.26)	<0.001	1.61(1.34,1.94)	<0.001	1.48(1.19,1.85)	<0.001
MDS≥3	4.35(3.72,5.09)	<0.001	2.02(1.67,2.44)	<0.001	1.83(1.46,2.30)	<0.001

Data are presented as OR (95% CI). Model I adjusted for age, sex and race/ethnicity. Model II adjusted for age, sex, race/ethnicity, education levels, BMI, smoking, HbA1c, TC, hypertension, DM, calcium, phosphorus, phosphorus intake, calcium intake, magnesium intake, CDAI, vitmain D intake, physical activity and poverty income ratio. MDS, Magnesium depletion score; OA, osteoarthritis.

**Figure 2:**
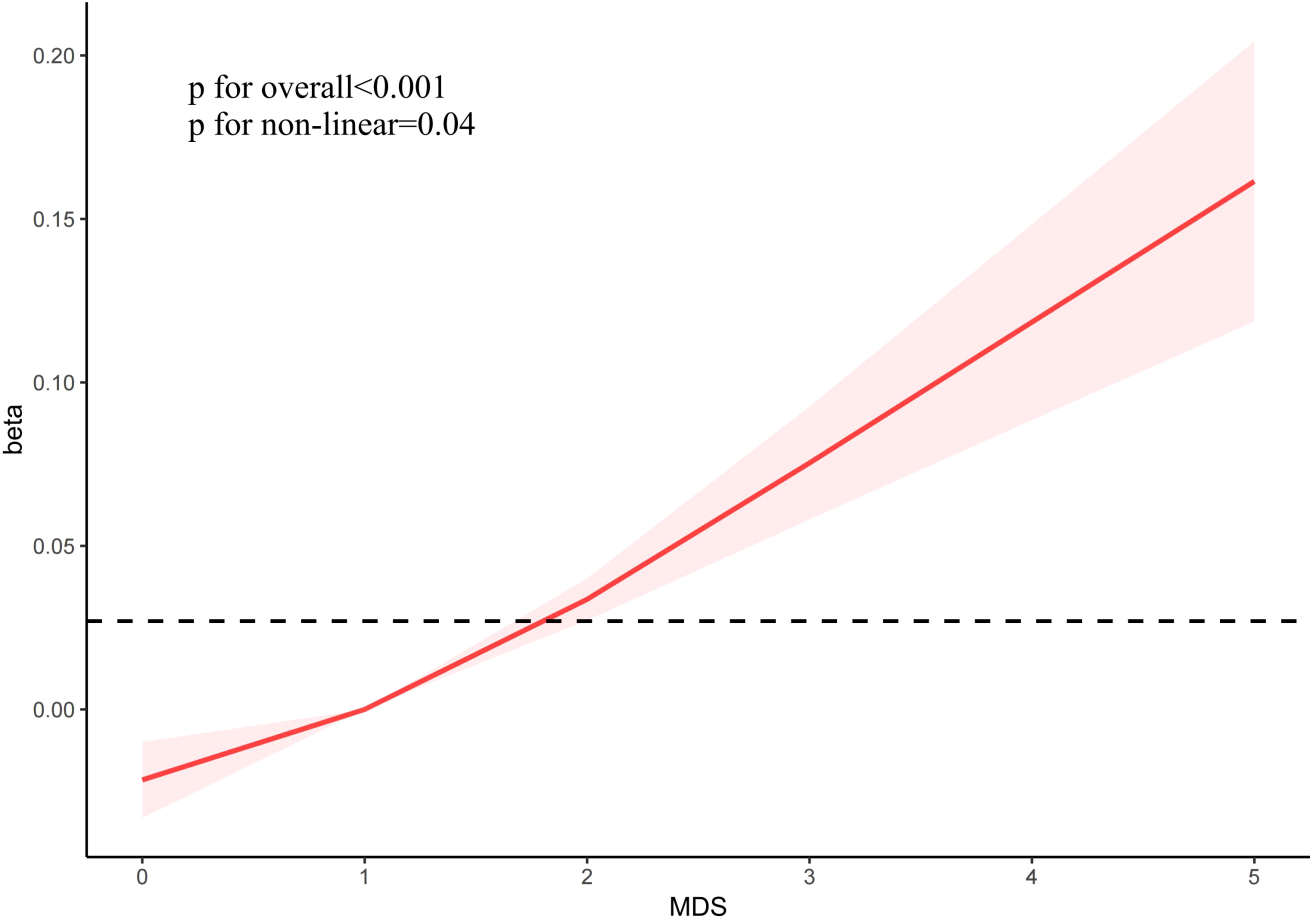
The correlation between MDS and the incidence of OA.

**Figure 2** showed a non-linear positive link between MDS and the incidence of OA (p for overall < 0.001, p for non-linear = 0.04). Moreover, in various subgroups such as age, sex, smoking, and DM, positive relationships were observed between MDS and the incidence of OA (**Supplementary Figure S1**).

**Figure 3:**
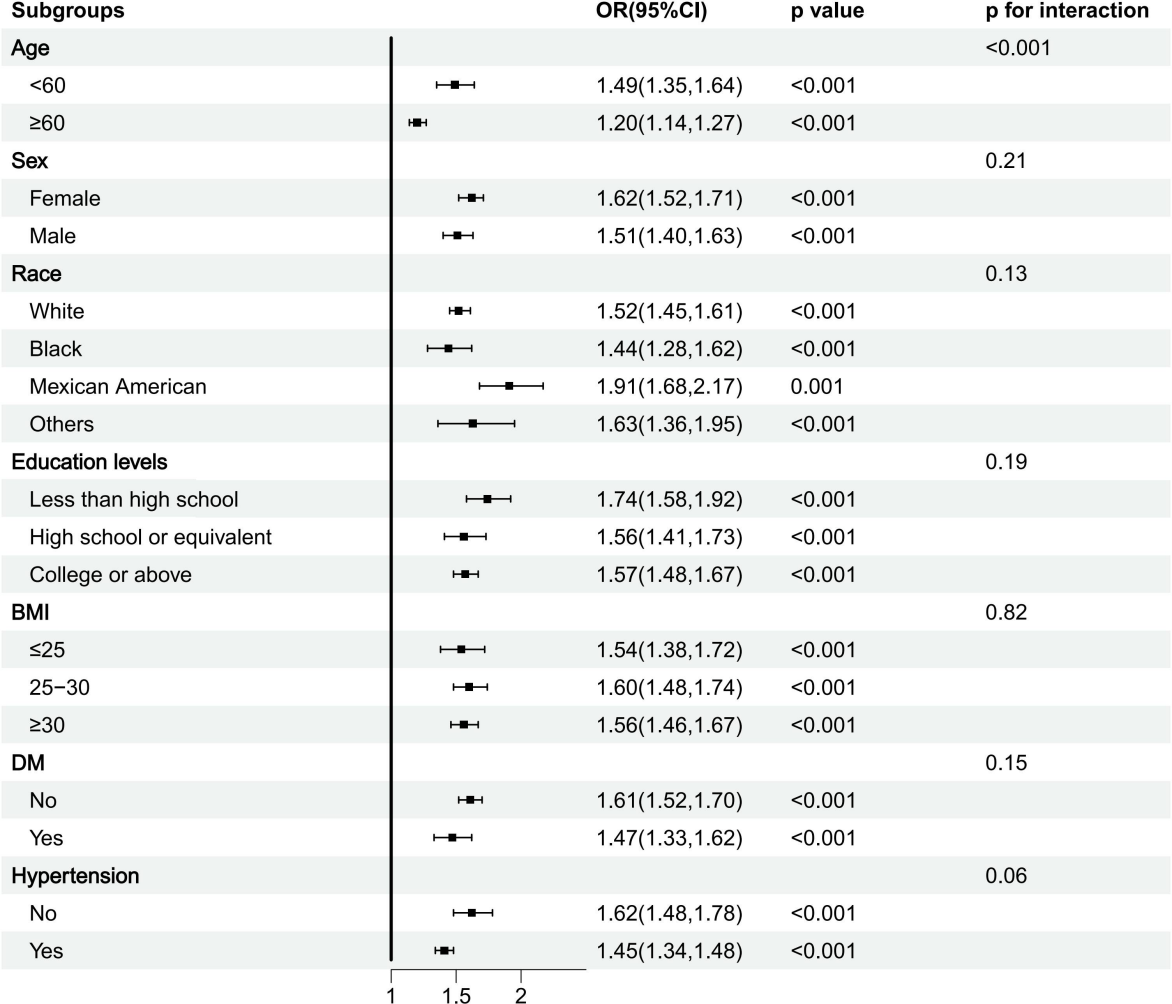
Subgroup analysis of MDS with the incidence of OA.

Subgroup analysis showed that the results were significant and stable. This relationship is slightly weakened in elderly people over 60 years old but still statistically significant (*P*<0.001) (**Figure 3**).

### The link between MDS and mortality among OA individuals

Second, we excluded individuals without OA (n = 16,138) and those missing data on follow-up information (n = 6). Consequently, the sample comprised 3,250 participants. A total of 630 deaths from all causes and 172 fatalities due to CVDs were recorded until December 31, 2019 (**Figure 1**). 3,250 participants were involved in the cohort study, including 630 all-cause deaths and 172 CVDs. As illustrated in **Supplementary Table S2**, the data were categorized into four groups based on MDS levels. In comparison to the MDS≥3 group, individuals in the MDS=0 category exhibit a greater percentage of all-cause mortality (30.46%vs.6.79%) and CVDs (9.65%vs.1.93%) (*P*<0.001). Individuals in the MDS≥3 cohort tend to be older (70.29 ± 0.51vs.55.77 ± 0.46) and have a higher likelihood of being female (74.09%vs.66.60%) and white (88.20%vs.74.72%), as well as a greater prevalence of hypertension (89.00%vs.46.98%) and diabetes (35.96%vs.21.18%) (*P*<0.001). The differences were also found between laboratory examination measurements (HbA1c, TC and phosphorus) and dietary factors (phosphorus intake, calcium intake and magnesium intake) (*P* < 0.001).

**Figure 4:**
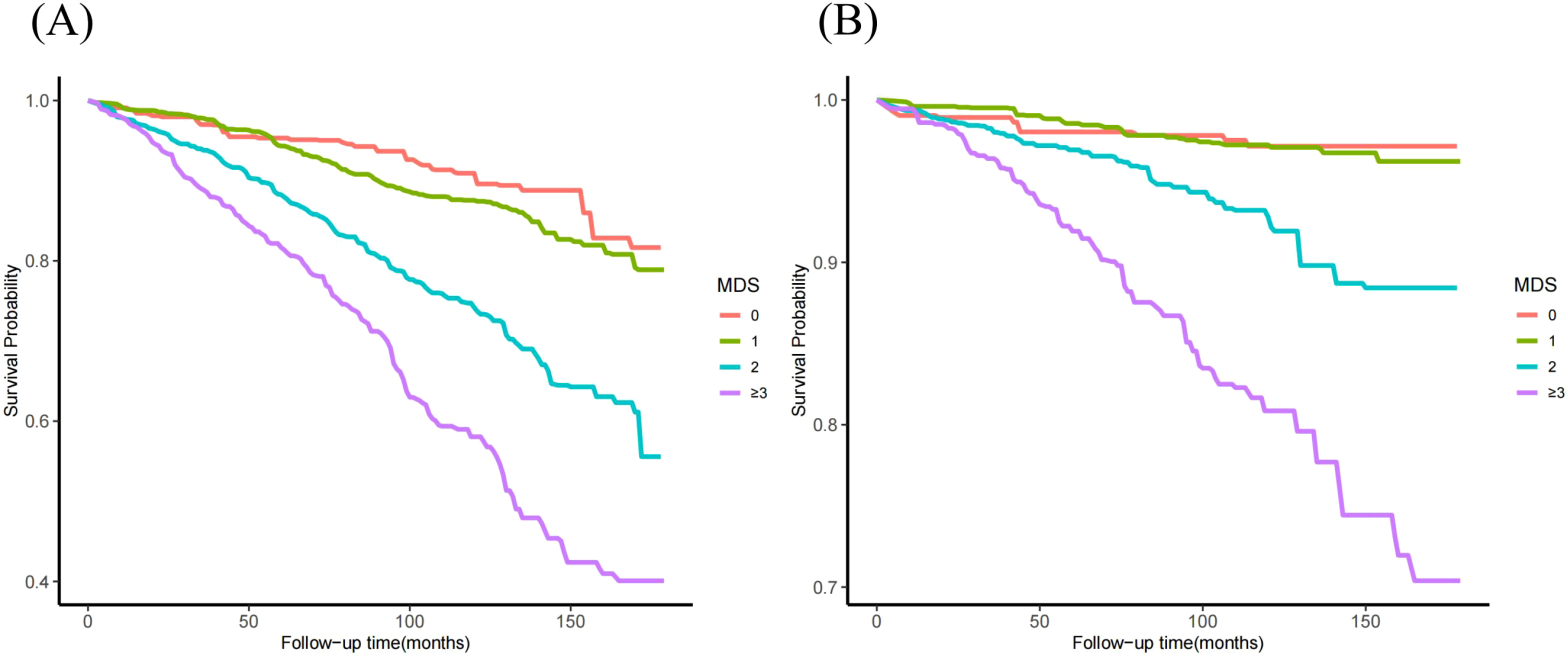
Kaplan-Meier survival analysis curves for mortality in different MDS groups. **(A)** all-cause mortality; **(B)** cardiovascular mortality.

Weighted Kaplan-Meier curves along with log-rank tests were employed to analyze the differences in cumulative survival across various MDS groups. The group with MDS≥3 has the highest all-cause mortality rate. The group with MDS=1 or MDS=2 has a moderate survival rate, while the group with MDS=0 have the highest survival rate (long-rank *P*<0.001) (**Figure 4A**). Similar death patterns are also reflected in CVDs (**Figure 4B**).

A positive association was observed between MDS and all-cause mortality with an HR of 1.44 (95%CI:1.27-1.62) (**Table 4**) and cardiovascular mortality with an HR of 1.64 (95%CI: 1.33-2.02) (**Table 5**) in adjusted cox regression analysis. Besides, MDS was also positively related to all-cause mortality [MDS≥3 vs. MDS=0, HR =2.56 (1.49-4.41, *P*<0.001)] and CVDs [MDS≥3 vs. MDS=0, HR =3.00 (1.13-7.98, *P*=0.01)] as a categorical variable. Sensitivity analysis also confirmed this finding after excluding OA patients who died within two years (**Supplementary Table S4**).

**Table 4:** Cox regression analysis on the association between MDS and all-cause mortality.

	Non-adjusted model		Model I		Model II	
	HR [95% CI]	*P* value	HR [95% CI]	*P* value	HR [95% CI]	*P* value
Continuous MDS	1.69(1.54,1.85)	<0.001	1.48(1.35,1.61)	<0.001	1.44(1.27,1.62)	<0.001
MDS=0	Reference	–	Reference	–	Reference	–
MDS=1	1.28(0.77,2.13)	0.35	0.92(0.56,1.51)	0.75	0.98(0.51,1.86)	0.95
MDS=2	2.89(1.84,4.51)	<0.001	1.82(1.18,2.82)	0.01	1.71(1.13,2.95)	0.03
MDS≥3	5.05(3.16,8.05)	<0.001	2.81(1.81,4.38)	<0.001	2.56(1.49,4.41)	<0.001

Data are presented as HR (95% CI). Model I adjusted for age, sex and race/ethnicity. Model II adjusted for age, sex, race/ethnicity, education levels, BMI, smoking, HbA1c, TC, hypertension, DM, calcium, phosphorus, phosphorus intake, calcium intake, magnesium intake, CDAI, vitmain D, physical activity and poverty income ratio. MDS, Magnesium depletion score; OA, osteoarthritis.

**Table 5:** Cox regression analysis on the association between MDS and cardiovascular mortality.

	Non-adjusted model		Model I		Model II	
	HR [95% CI]	*P* value	HR [95% CI]	*P* value	HR [95% CI]	*P* value
Continuous MDS	1.98(1.63,2.41)	<0.001	1.76(1.46,2.13)	<0.001	1.64(1.33, 2.02)	<0.001
MDS=0	Reference					
MDS=1	0.91(0.29, 2.80)	0.86	0.61(0.21,1.83)	0.38	0.68(0.22, 2.10)	0.51
MDS=2	2.53(0.89, 7.21)	0.08	1.53(0.57,4.13)	0.40	1.34(0.48, 3.73)	0.57
MDS≥3	6.43(2.28,18.08)	<0.001	3.47(1.35,8.95)	0.01	3.00(1.13, 7.98)	0.01

Data are presented as HR (95% CI). Model I adjusted for age, sex and race/ethnicity. Model II adjusted for age, sex, race/ethnicity, education levels, BMI, smoking, HbA1c, TC, hypertension, DM, calcium, phosphorus, phosphorus intake, calcium intake, magnesium intake, CDAI, vitmain D intake, physical activity and poverty income ratio. MDS, Magnesium depletion score; OA, osteoarthritis.

**Figure 5:**
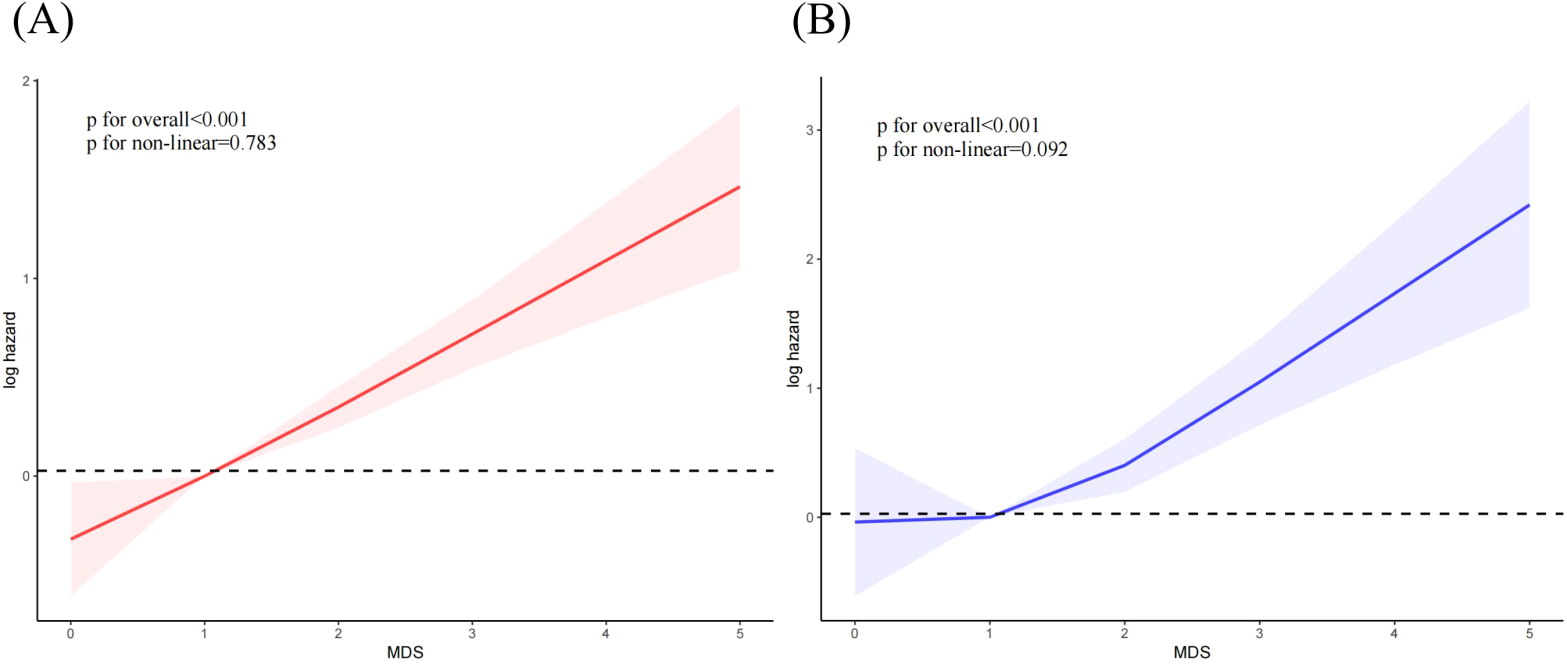
The correlation between MDS and mortality among OA individuals. **(A)** all-cause mortality; **(B)** cardiovascular mortality.

**Figure 5** showed a linear positive correlation between MDS and all-cause mortality (p for overall < 0.001, p for non-linear = 0.783) and CVDs (*P* for overall < 0.001, *P* for non-linear = 0.092) among OA individuals.

Subgroup analysis indicated that the results were significant and stable in most subgroups. Notably, this relationship was only significant in individuals aged 60 and above in the age subgroup (*P*<0.001) (**Supplementary Figure S2**).

## Discussion

Our study indicates a positive correlation between MDS and the incidence of OA among US middle aged and elderly people. Subgroup analysis indicated that the results were significant and stable in most subgroups. Moreover, there was a positive correlation between MDS and the mortality of OA.

Magnesium plays an important role in inflammatory diseases and has good anti-inflammatory effects. Research has found that giving mice a high magnesium diet can reduce levels of inflammatory factors (IL-1β, TNF-α and IL-6) in the body, alleviate joint inflammation and joint damage. Mechanistically, magnesium increases the number of Foxp3^+^Treg cells in an IL-10-dependent manner mediated by gut microbiota (24). The latest research has also found that magnesium can effectively reduce or even reverse the degeneration of cartilage tissue. Magnesium can enhance the proliferation and chondrogenic differentiation of bone marrow mesenchymal stem cells, and has the potential to promote joint cartilage regeneration. In addition, magnesium can also inhibit programmed cell death of chondrocytes, thereby protecting joint cartilage. For osteoclasts, magnesium can inhibit their generation and bone degradation functions (25). Therefore, magnesium has significant potential in the treatment of arthritis. The evaluation of MDS may have significant implications in OA.

In clinical practice, serum magnesium was often used to assess magnesium status. A study involving 2855 patients revealed that serum magnesium concentration was negatively linked to the incidence of imaging knee OA (26). Similarly, a meta-analysis showed that elevated serum magnesium levels correlate with a reduced incidence of OA, but this relationship is significantly affected by serum magnesium concentration (27). The possible reason is that serum magnesium may not comprehensively reflect the magnesium status of the body. Especially in cases of chronic magnesium deficiency, serum magnesium may still remain at normal levels due to the body’s compensation (28, 29). While serum magnesium is utilized in clinical practice, a clear correlation has yet to be established between serum magnesium concentrations and systemic magnesium levels or the concentrations found in particular tissues. In addition, the serum contain only holds 0.3% of the body’s total magnesium, and most of the rest remain in the organization (30). The National Institutes of Health (NIH) in the United States has pointed out that assessing magnesium levels is difficult due to the fact that most magnesium is present in cells or bones. When determining magnesium deficiency, this may lead to misleading blood test results. More than 80% of serum magnesium undergoes filtration and reabsorption in the kidneys. MDS incorporates pathophysiological factors influencing the renal reabsorption capability. Research has found that compared to serum magnesium, MDS has greater value in predicting the body’s magnesium status (16). Furthermore, the four risk factors included in MDS (current use of diuretics and PPIs, heavy alcohol consumption, and kidney function) are easily assessable in clinical practice.

Evaluating urinary magnesium offers an additional approach for determining the body’s magnesium levels, but it is easily influenced by diet, medication, and kidney disease (31). And for people with limited mobility, monitoring 24-hour urinary magnesium is difficult to widely implement (32). The evaluation of magnesium tolerance is considered the benchmark for determining the magnesium levels within the body. However, its application is severely limited due to the complexity of its operation and its unsuitability for patients with renal dysfunction (33). MDS is a practical tool for assessing the magnesium status of the body (16). Therefore, exploring its relationship with diseases may provide important guidance for clinical practice.

Magnesium deficiency is very common in middle-aged and elderly populations, with reasons including insufficient magnesium intake and increased excretion caused by various medications (34). In the cross-sectional study, our study found that this relationship between MDS and the incidence of OA is slightly weakened in elderly people over 60 years old but still statistically significant (*P*<0.001). We consider that the difference in magnesium deficiency levels between non-OA and OA patients in the elderly population may gradually narrow. This may be one of the possible reasons for the occurrence of this result. Additionally, this link between MDS and mortality was only significant in individuals aged 60 and above in the cohort study. Elderly OA patients often have multiple comorbidities and a higher proportion of diuretic use, often accompanied by renal dysfunction. This may partially explain the significant relationship between MDS and all-cause mortality in elderly OA patients. Therefore, in OA patients over 60 years old, MDS may be a better tool for assessing prognosis. Furthermore, we also observed that MDS is significantly associated with the incidence of OA and the death of OA patients in the subgroups of hypertension and diabetes (*P*<0.001). However, no significant interaction was observed between the two subgroups. This shows that the effect of magnesium deficiency on OA is not affected by hypertension and diabetes.

MDS consists of four elements that influence renal reabsorption: alcohol intake, the usage of diuretics, the use of PPIs, and kidney function (16). PPI mainly causes magnesium deficiency in the body by affecting the intestinal reabsorption of magnesium (35). Some patients who use PPIs in clinical practice may experience hypomagnesemia, but this phenomenon can be alleviated by supplementing magnesium or discontinuing medication. Recent studies have shown that the use of PPIs may accelerate the progression of OA (36), possibly through drug-induced magnesium deficiency, which is consistent with our research findings. Alcohol is deemed crucial for magnesium excretion. The mechanism may be that excessive alcohol use cause renal tubular damage, resulting in an increase in urinary magnesium (37). Magnesium deficiency may trigger systemic inflammatory reactions and promote the production of inflammatory mediators by chondrocytes and synovial cells, ultimately damaging the synthesis of articular cartilage (38). Omeprazole is a classic proton pump inhibitor. Omeprazole may cause an increase in segmental pH value in the small intestine after inhibiting gastric acid secretion, leading to a decrease in Mg^2+^ dissolution and reabsorption (39). Another presumed mechanism for the diminished absorption of magnesium by intestinal epithelial cells involves the PPIs-induced inhibition of transient receptor potential melastatin-6 (TRPM6) and TRPM7 channels. These factors lead to magnesium deficiency, which triggers joint inflammation and promotes the progression of OA (40). The kidneys are the most important organ for magnesium reabsorption, and the body’s magnesium balance depends on the involvement of the kidneys (41). Many diuretics also act on the renal tubules and inhibit magnesium reabsorption (42). For example, thiazide diuretics can act on the renal tubules, significantly reducing the reabsorption of magnesium in the body and serum magnesium concentration, indirectly inducing joint inflammation and the occurrence of OA (43).

In addition, we found that the use of diuretics and decreased renal function are risk factors for mortality in OA patients. Previous studies have shown that renal dysfunction is an independent risk factor for mortality (44). Besides, middle-aged and elderly people are often accompanied by other diseases, including cardiovascular disease, hypertension, diabetes, etc. It is understandable that those who take diuretics face an increased risk of mortality.

Despite the advantage of NHANES large sample research, our study still has some limitations. First, we cannot identify a causal association due to the type of cross-sectional study. Second, the results from the study mainly apply to the American population. Third, while the study utilizes MDS, the reliance on indirect markers (diuretic and PPI use) may not fully capture magnesium deficiency. we are unable to compare their advantages and disadvantages due to the lack of serum magnesium and urinary magnesium. Large prospective studies are needed in the future to further validate the role of MDS in evaluating magnesium deficiency. Finally, there may still be other confounding factors affecting the results although many variables are included.

## Conclusion

MDS is positively correlated with the incidence and mortality of OA. Optimizing the nutritional status of magnesium may bring benefits to OA patients.

## Data Availability

Publicly available datasets were analyzed in this study. This data can be found here: https://www.cdc.gov/nchs/nhanes/.

## Supplementary material

Supplementary Figure 1 The correlation between MDS and the incidence of OA in different subgroups. **(A)** Age; **(B)** Sex; **(C)** Smoke; **(D)** DM.

Supplementary Figure 2 Subgroup analysis of MDS with mortality among OA individuals. **(A)** all-cause mortality; **(B)** cardiovascular mortality.
