# Supplementary figures and images for: Associations of magnesium depletion score with the incidence and mortality of osteoarthritis: a nationwide study
Source: Front Immunol. 2025 Feb 28;16:1512293. doi: 10.3389/fimmu.2025.1512293 (PMC11907003; doi:10.3389/fimmu.2025.1512293)

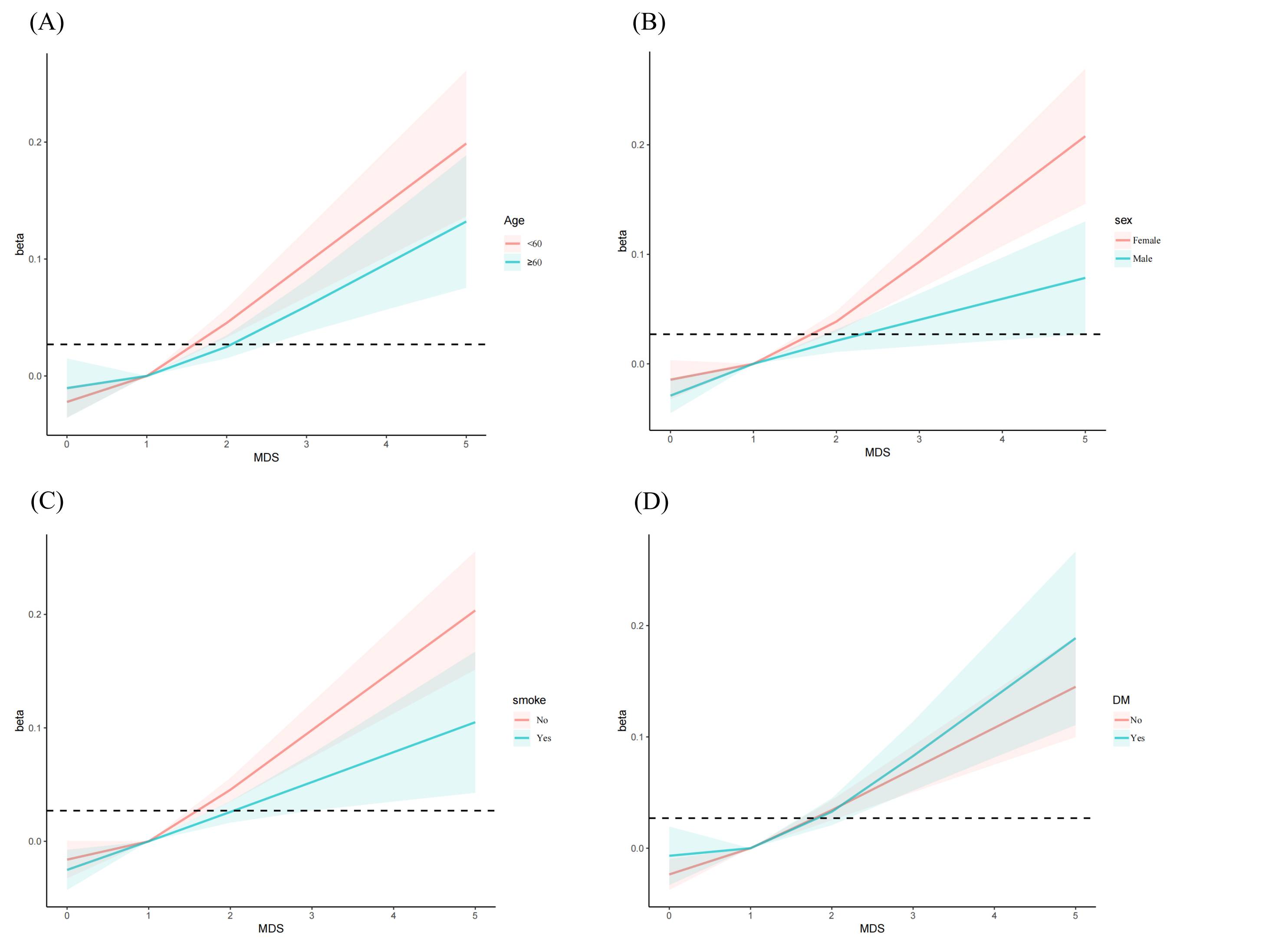

Supplement: Supplementary Figure 1 — The correlation between MDS and the incidence of OA in different subgroups. (A) Age; (B) Sex; (C) Smoke; (D) DM. [file Image1.jpeg]

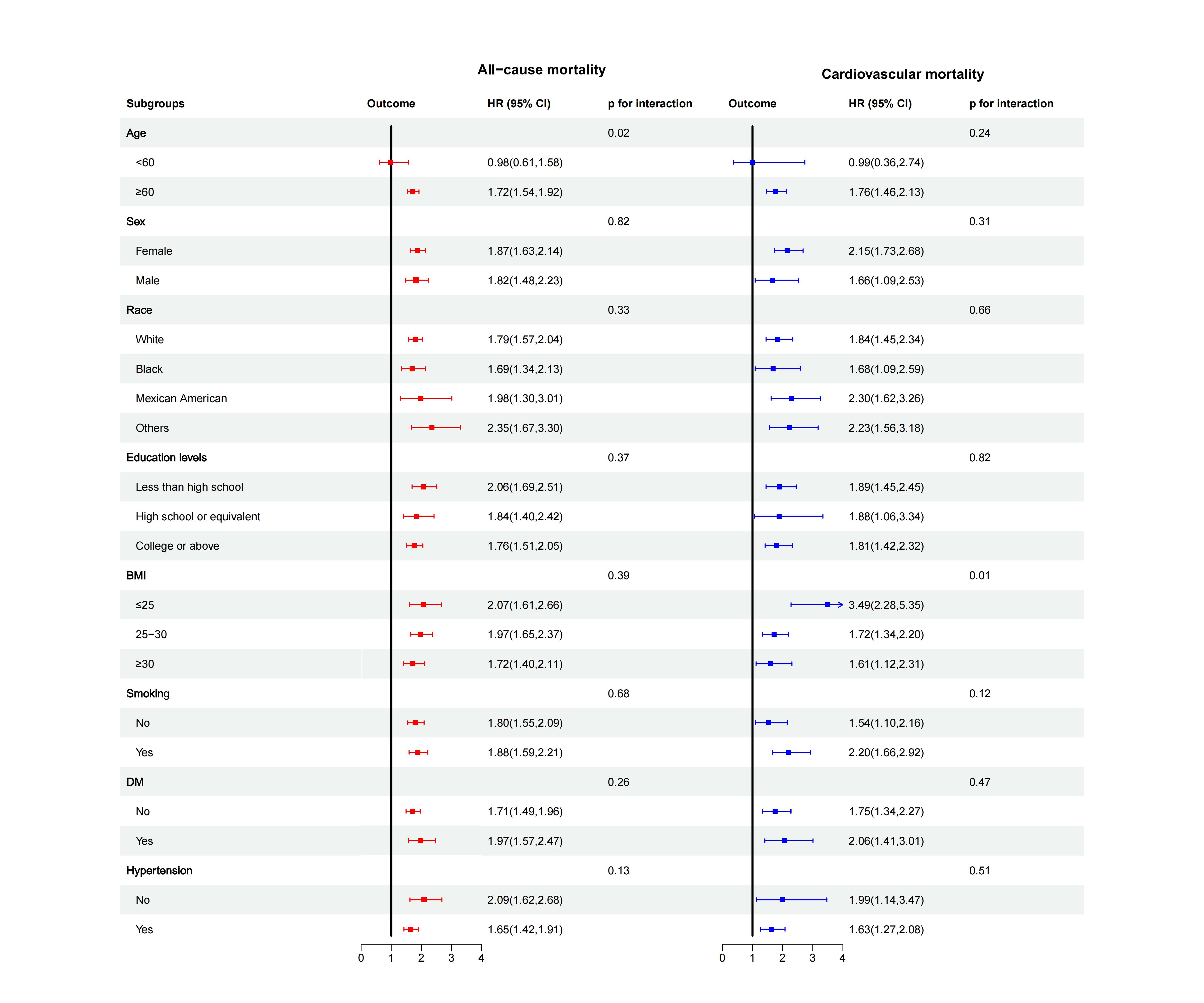

Supplement: Supplementary Figure 2 — Subgroup analysis of MDS with mortality among OA individuals. (A) all-cause mortality; (B) cardiovascular mortality. [file Image2.jpeg]
