# Supplementary material for: Associations of magnesium depletion score with the incidence and mortality of osteoarthritis: a nationwide study
Source: Front Immunol. 2025 Feb 28;16:1512293. doi: 10.3389/fimmu.2025.1512293 (PMC11907003; doi:10.3389/fimmu.2025.1512293)
Supplement: Supplementary file 3 [file DataSheet1.zip › Data Sheet 1/Table S1.DOCX]

**Table S1. Clinical characteristics of the the total population.**

| **Variables** | **Overall** | **MDS=0** | **MDS=1** | **MDS=2** | **MDS≥3** | ***P* value** |
| --- | --- | --- | --- | --- | --- | --- |
| **Age, %** | 57.22±0.16 | 50.61±0.17 | 56.89±0.19 | 62.77±0.27 | 68.65±0.35 | <0.001 |
| **Sex, %** |  |  |  |  |  | <0.001 |
| Female | 52.09(49.33,54.84) | 53.15(51.67,54.63) | 49.51(48.23,50.78) | 51.39(49.35,53.43) | 61.75(59.34,64.15) |  |
| Male | 47.91(45.24,50.58) | 46.85(45.37,48.33) | 50.49(49.22,51.77) | 48.61(46.57,50.65) | 38.25(35.85,40.66) |  |
| **Race/ethnicity, %** |  |  |  |  |  | <0.001 |
| White | 73.30(67.44,79.15) | 60.51(57.51,63.50) | 77.03(74.58,79.47) | 81.09(78.89,83.30) | 82.45(80.06,84.84) |  |
| Black | 9.46(8.46,10.45) | 11.31(9.87,12.75) | 8.45(7.24, 9.67) | 8.50(7.11, 9.89) | 9.84(8.17,11.51) |  |
| Mexican | 6.32(5.34, 7.30) | 11.59(9.65,13.53) | 4.79(3.89, 5.70) | 3.15(2.36, 3.95) | 2.41(1.60, 3.23) |  |
| Others | 10.92(9.95,11.89) | 16.59(14.99,18.20) | 9.72(8.49,10.95) | 7.26(5.98, 8.54) | 5.30(4.24, 6.36) |  |
| **Education levels, %** |  |  |  |  |  | <0.001 |
| Less than high school | 15.36(14.15,16.57) | 17.59(16.00,19.19) | 13.66(12.29,15.03) | 14.68(13.43,15.93) | 17.05(14.65,19.44) |  |
| High school or equivalent | 23.53(21.75,25.31) | 23.11(21.49,24.73) | 23.23(21.74,24.72) | 23.90(22.02,25.79) | 25.55(22.90,28.19) |  |
| College or above | 61.07(57.35,64.78) | 59.29(56.94,61.65) | 63.11(60.89,65.33) | 61.42(59.01,63.83) | 57.41(54.43,60.39) |  |
| **BMI, kg/m2** | 29.30±0.08 | 29.25±0.12 | 28.84±0.11 | 29.66±0.15 | 30.77±0.22 | <0.001 |
| **HbA1c, %** | 5.78±0.01 | 5.77±0.02 | 5.70±0.01 | 5.84±0.02 | 6.01±0.03 | <0.001 |
| **TC, mmol/L** | 5.17±0.01 | 5.21±0.02 | 5.23±0.02 | 5.12±0.03 | 4.94±0.04 | <0.001 |
| **Serum calcium, mmol/L** | 2.35±0.00 | 2.34±0.00 | 2.35±0.00 | 2.36±0.00 | 2.36±0.00 | <0.001 |
| **Serum phosphorus, mmol/L** | 1.20±0.00 | 1.19±0.00 | 1.19±0.00 | 1.21±0.00 | 1.22±0.01 | <0.001 |
| **Magnesium intake, mg** | 305.46±1.92 | 308.69±2.96 | 315.53±2.71 | 299.51±3.25 | 262.42±3.84 | <0.001 |
| **Calcium intake, mg** | 940.36±7.09 | 977.17±10.06 | 956.74± 9.92 | 900.90±12.95 | 830.45±17.34 | <0.001 |
| **Phosphorus intake, mg** | 1360.89±7.11 | 1397.71±11.96 | 1395.57±10.64 | 1313.97±12.52 | 1185.31±17.19 | <0.001 |
| **Vitmain D intake, mcg** | 4.67±0.07 | 4.71±0.10 | 4.72±0.10 | 4.58±0.18 | 4.57±0.17 | 0.81 |
| **CDAI** | 0.75±0.06 | 0.98±0.09 | 0.83±0.08 | 0.56±0.10 | 0.00±0.12 | <0.001 |
| **Physical activity, %** |  |  |  |  |  | <0.001 |
| No | 53.00(50.22,55.78) | 53.89(51.50,56.29) | 48.46(46.48,50.44) | 55.81(53.45,58.18) | 63.95(60.97,66.94) |  |
| Yes | 47.00(43.81,50.19) | 46.11(43.71,48.50) | 51.54(49.56,53.52) | 44.19(41.82,46.55) | 36.05(33.06,39.03) |  |
| **Poverty income ratio, %** |  |  |  |  |  | <0.001 |
| ≤1.30 | 15.50(14.27,16.72) | 18.88(17.13,20.62) | 15.19(13.76,16.63) | 15.19(13.51,16.88) | 18.52(16.20,20.83) |  |
| 1.31–3.49 | 32.27(30.15,34.39) | 34.37(32.09,36.66) | 33.11(31.01,35.20) | 34.71(32.58,36.84) | 41.69(38.21,45.17) |  |
| ≥3.50 | 45.55(42.00,49.10) | 46.75(43.68,49.82) | 51.70(49.16,54.25) | 50.10(47.38,52.81) | 39.79(35.78,43.81) |  |
| **Smoking, %** |  |  |  |  |  | <0.001 |
| No | 52.74(50.02,55.46) | 55.45(53.25,57.66) | 53.16(51.14,55.19) | 49.63(47.53,51.72) | 48.91(45.97,51.84) |  |
| Yes | 47.22(44.19,50.26) | 44.55(42.34,46.75) | 46.84(44.81,48.86) | 50.37(48.28,52.47) | 51.09(48.16,54.03) |  |
| **Hypertension, %** |  |  |  |  |  | <0.001 |
| No | 50.78(47.70,53.85) | 66.61(64.94,68.28) | 55.75(53.74,57.76) | 33.64(31.62,35.67) | 13.36(11.25,15.46) |  |
| Yes | 49.22(46.55,51.88) | 33.39(31.72,35.06) | 44.25(42.24,46.26) | 66.36(64.33,68.38) | 86.64(84.54,88.75) |  |
| **DM, %** |  |  |  |  |  | <0.001 |
| No | 80.86(76.34,85.39) | 83.96(82.75,85.17) | 84.37(83.21,85.52) | 76.21(74.51,77.91) | 65.89(62.86,68.92) |  |
| Yes | 19.05(17.92,20.19) | 16.04(14.83,17.25) | 15.63(14.48,16.79) | 23.79(22.09,25.49) | 34.11(31.08,37.14) |  |
| **OA, %** |  |  |  |  |  | <0.001 |
| No | 80.95(76.77,85.14) | 88.89(87.64,90.14) | 81.97(80.59,83.35) | 74.14(72.37,75.91) | 64.76(62.22,67.30) |  |
| Yes | 19.05(17.63,20.46) | 11.11( 9.86,12.36) | 18.03(16.65,19.41) | 25.86(24.09,27.63) | 35.24(32.70,37.78) |  |

Continuous data were presented as the mean±SEM, category data were presented as the proportion and 95% confidence interval. SEM, Standard Error of the Mean; MDS, Magnesium depletion score; BMI, body mass index; HbA1c, glycosylated hemoglobin; TC, total cholesterol; DM, diabetes mellitus; CDAI, composite dietary antioxidant index; OA, osteoarthritis.
