# Supplementary material for: Associations of magnesium depletion score with the incidence and mortality of osteoarthritis: a nationwide study
Source: Front Immunol. 2025 Feb 28;16:1512293. doi: 10.3389/fimmu.2025.1512293 (PMC11907003; doi:10.3389/fimmu.2025.1512293)
Supplement: Supplementary file 3 [file DataSheet1.zip › Data Sheet 1/Table S2.DOCX]

**Table S2. Clinical characteristics of the OA participants.**

| **Variables** | **Overall** | **MDS=0** | **MDS=1** | **MDS=2** | **MDS≥3** | ***P* value** |
| --- | --- | --- | --- | --- | --- | --- |
| **Age, %** | 63.59±0.27 | 55.77±0.46 | 62.17±0.36 | 66.56±0.43 | 70.29±0.51 | <0.001 |
| **Sex, %** |  |  |  |  |  | <0.001 |
| Female | 64.83(59.57,70.09) | 66.60(61.62,71.57) | 60.99(57.26,64.73) | 63.51(59.81,67.22) | 74.09(69.87,78.32) |  |
| Male | 35.17(31.94,38.39) | 33.40(28.43,38.38) | 39.01(35.27,42.74) | 36.49(32.78,40.19) | 25.91(21.68,30.13) |  |
| **Race/ethnicity, %** |  |  |  |  |  | <0.001 |
| White | 84.17(76.58,91.75) | 74.72(70.48,78.96) | 84.93(82.07,87.78) | 86.77(84.59,88.95) | 88.20(85.67,90.74) |  |
| Black | 6.09( 5.09, 7.09) | 8.44(6.37,10.51) | 5.68(4.43, 6.93) | 4.86(3.54, 6.17) | 6.58(4.65, 8.51) |  |
| Mexican | 2.55( 1.93, 3.17) | 4.56(2.95,6.18) | 2.36(1.57,3.16) | 2.06(1.36,2.77) | 1.62(0.85,2.39) |  |
| Others | 7.20( 6.07, 8.32) | 12.27(8.73,15.82) | 7.03(5.02, 9.04) | 6.31(4.39, 8.23) | 3.59(2.26, 4.93) |  |
| **Education levels, %** |  |  |  |  |  | 0.38 |
| Less than high school | 12.10(10.54,13.66) | 9.72( 6.92,12.52) | 11.67( 9.28,14.05) | 13.16(10.75,15.58) | 13.91(11.09,16.73) |  |
| High school or equivalent | 23.23(20.26,26.20) | 24.30(18.34,30.27) | 22.27(19.32,25.21) | 22.45(19.11,25.78) | 25.67(20.89,30.44) |  |
| College or above | 64.66(59.15,70.16) | 65.98(59.52,72.44) | 66.07(62.33,69.81) | 64.39(60.47,68.31) | 60.43(55.64,65.22) |  |
| **BMI, kg/m2** | 30.78±0.20 | 30.29±0.43 | 30.48±0.32 | 30.68±0.33 | 32.18±0.43 | 0.004 |
| **HbA1c, %** | 5.84±0.02 | 5.88±0.06 | 5.76±0.03 | 5.82±0.03 | 6.01±0.04 | <0.001 |
| **TC, mmol/L** | 5.13±0.02 | 5.16±0.05 | 5.24±0.05 | 5.06±0.05 | 4.96±0.06 | 0.002 |
| **Serum calcium, mmol/L** | 2.36±0.00 | 2.34±0.01 | 2.36±0.00 | 2.36±0.00 | 2.36±0.01 | 0.12 |
| **Serum phosphorus, mmol/L** | 1.22±0.00 | 1.22±0.01 | 1.21±0.01 | 1.23±0.01 | 1.24±0.01 | 0.02 |
| **Magnesium intake, mg** | 294.33±3.49 | 296.05±8.43 | 313.37±5.48 | 287.15±5.82 | 260.51±6.17 | <0.001 |
| **Calcium intake, mg** | 913.56±14.49 | 977.74±30.76 | 961.98±28.88 | 878.35±22.78 | 791.54±24.05 | <0.001 |
| **Phosphorus intake, mg** | 1297.32±16.03 | 1319.46±35.26 | 1384.20±31.80 | 1253.82±23.54 | 1145.81±25.28 | <0.001 |
| **Vitmain D intake, mcg** | 4.54±0.13 | 4.66±0.27 | 4.75±0.23 | 4.34±0.25 | 4.32±0.24 | 0.52 |
| **CDAI** | 0.65±0.10 | 0.50±0.22 | 1.09±0.17 | 0.52±0.21 | 0.04±0.17 | <0.001 |
| **Physical activity, %** |  |  |  |  |  | <0.001 |
| No | 54.85(50.17,59.53) | 52.18(45.38,58.98) | 48.20(44.20,52.21) | 59.46(55.84,63.09) | 65.31(60.95,69.68) |  |
| Yes | 45.15(40.86,49.44) | 47.82(41.02,54.62) | 51.80(47.79,55.80) | 40.54(36.91,44.16) | 34.69(30.32,39.05) |  |
| **Poverty income ratio, %** |  |  |  |  |  | 0.06 |
| ≤1.30 | 14.40(12.58,16.23) | 17.51(12.97,22.05) | 13.42(11.00,15.84) | 15.34(12.56,18.11) | 17.75(14.32,21.17) |  |
| 1.31–3.49 | 33.64(30.32,36.95) | 32.05(26.08,38.01) | 34.99(30.86,39.11) | 36.45(32.02,40.89) | 41.58(36.14,47.03) |  |
| ≥3.50 | 45.48(40.52,50.43) | 50.44(43.47,57.41) | 51.59(46.73,56.45) | 48.21(43.25,53.17) | 40.67(34.83,46.51) |  |
| **Smoking, %** |  |  |  |  |  | 0.43 |
| No | 48.60(44.47,52.73) | 45.79(38.95,52.64) | 49.13(44.68,53.58) | 47.48(43.28,51.68) | 52.54(47.41,57.67) |  |
| Yes | 51.33(46.32,56.33) | 54.21(47.36,61.05) | 50.87(46.42,55.32) | 52.52(48.32,56.72) | 47.46(42.33,52.59) |  |
| **Hypertension, %** |  |  |  |  |  | <0.001 |
| No | 35.90(32.11,39.68) | 53.02(47.69,58.35) | 44.75(40.69,48.82) | 27.54(23.86,31.22) | 11.00( 7.92,14.08) |  |
| Yes | 64.10(58.94,69.27) | 46.98(41.65,52.31) | 55.25(51.18,59.31) | 72.46(68.78,76.14) | 89.00(85.92,92.08) |  |
| **DM, %** |  |  |  |  |  | <0.001 |
| No | 76.43(70.36,82.49) | 78.82(73.80,83.84) | 81.84(79.00,84.68) | 74.79(71.47,78.12) | 64.04(59.61,68.48) |  |
| Yes | 23.57(21.00,26.15) | 21.18(16.16,26.20) | 18.16(15.32,21.00) | 25.21(21.88,28.53) | 35.96(31.52,40.39) |  |
| **All-cause mortality, %** |  |  |  |  |  | <0.001 |
| No | 85.28(78.61,91.95) | 93.21(90.29,96.14) | 91.03(88.84,93.23) | 81.62(78.56,84.69) | 69.54(64.83,74.24) |  |
| Yes | 14.72(12.84,16.61) | 6.79( 3.86, 9.71) | 8.97( 6.77,11.16) | 18.38(15.31,21.44) | 30.46(25.76,35.17) |  |
| **Cardiovascular mortality, %** |  |  |  |  |  | <0.001 |
| No | 96.23(88.83,103.62) | 98.07(96.17,99.97) | 98.24(97.40,99.09) | 95.75(94.31,97.20) | 90.35(87.86,92.83) |  |
| Yes | 3.77( 3.01, 4.54) | 1.93(0.03, 3.83) | 1.76(0.91, 2.60) | 4.25(2.80, 5.69) | 9.65(7.17,12.14) |  |

Continuous data were presented as the mean±SEM, category data were presented as the proportion and 95% confidence interval. SEM, Standard Error of the Mean; MDS, Magnesium depletion score; BMI, body mass index; HbA1c, glycosylated hemoglobin; TC, total cholesterol; CDAI, composite dietary antioxidant index; DM, diabetes mellitus.
