# Supplementary material for: Associations of magnesium depletion score with the incidence and mortality of osteoarthritis: a nationwide study
Source: Front Immunol. 2025 Feb 28;16:1512293. doi: 10.3389/fimmu.2025.1512293 (PMC11907003; doi:10.3389/fimmu.2025.1512293)
Supplement: Supplementary file 3 [file DataSheet1.zip › Data Sheet 1/Table S3.DOCX]

**Table S3. Unweighted logistic regression analysis on the association between MDS and OA in sensitivity analysis.**

|  | **Non-adjusted model** |  | **Model I** |  | **Model II** |  |
| --- | --- | --- | --- | --- | --- | --- |
|  | **OR [95% CI]** | ***P* value** | **OR [95% CI]** | ***P* value** | **OR [95% CI]** | ***P* value** |
| **Continuous MDS** | 1.60(1.54,1.66) | <0.001 | 1.27(1.22,1.33) | <0.001 | 1.23(1.18,1.29) | <0.001 |
| **MDS=0** | Reference | - | Reference | - | Reference | - |
| **MDS=1** | 1.87(1.68,2.08) | <0.001 | 1.32(1.18,1.48) | <0.001 | 1.23(1.08,1.39) | <0.001 |
| **MDS=2** | 3.07(2.74,3.44) | <0.001 | 1.73(1.52,1.96) | <0.001 | 1.58(1.38,1.82) | <0.001 |
| **MDS≥3** | 4.46(3.92,5.07) | <0.001 | 2.12(1.84,2.45) | <0.001 | 1.92(1.63,2.25) | <0.001 |

Data are presented as OR (95% CI). Model I adjusted for age, sex and race/ethnicity. Model II adjusted for age, sex, race/ethnicity, education levels, BMI, smoking, HbA1c, TC, hypertension, DM, calcium, phosphorus, phosphorus intake, calcium intake, magnesium intake, CDAI, vitmain D intake, physical activity and poverty income ratio. MDS, Magnesium depletion score; OA, osteoarthritis.
