# Supplementary material for: Associations of magnesium depletion score with the incidence and mortality of osteoarthritis: a nationwide study
Source: Front Immunol. 2025 Feb 28;16:1512293. doi: 10.3389/fimmu.2025.1512293 (PMC11907003; doi:10.3389/fimmu.2025.1512293)
Supplement: Supplementary file 3 [file DataSheet1.zip › Data Sheet 1/Table S4.DOCX]

**TableS4. Sensitive analysis to evaluate the association between MDS and mortality after excluding participants who died within 2 years.**

|  | **Non-adjusted model** |  | **Model I** |  | **Model II** |  |
| --- | --- | --- | --- | --- | --- | --- |
|  | **HR [95% CI]** | ***P* value** | **HR [95% CI]** | ***P* value** | **HR [95% CI]** | ***P* value** |
| **All-cause mortality** |  |  |  |  |  |  |
| **Continuous MDS** | 1.70(1.54,1.88) | <0.001 | 1.47(1.33,1.62) | <0.001 | 1.44(1.25,1.66) | <0.001 |
| **MDS=0** | Reference | - | Reference | - | Reference | - |
| **MDS=1** | 1.51(0.88,2.61) | 0.13 | 1.06(0.62,1.82) | 0.83 | 1.19(0.57,2.50) | 0.64 |
| **MDS=2** | 3.26(2.05,5.19) | <0.001 | 1.97(1.24,3.15) | 0.004 | 2.03(1.09,3.75) | 0.02 |
| **MDS≥3** | 5.87(3.64,9.48) | <0.001 | 3.08(1.90,5.01) | <0.001 | 2.95(1.57,5.54) | <0.001 |
| **Cardiovascular mortality** |  |  |  |  |  |  |
| **Continuous MDS** | 1.98(1.63,2.41) | <0.001 | 1.76(1.46,2.13) | <0.001 | 1.69(1.35, 2.10) | <0.001 |
| **MDS=0** | Reference |  |  |  |  |  |
| **MDS=1** | 1.50(0.46, 4.88) | 0.50 | 0.88(0.26, 2.91) | 0.83 | 1.00(0.29, 3.40) | 0.99 |
| **MDS=2** | 3.97(1.24,12.73) | 0.02 | 1.96(0.60, 6.41) | 0.27 | 1.78(0.52, 6.10) | 0.36 |
| **MDS≥3** | 11.92(3.94,36.10) | <0.001 | 4.89(1.58,15.07) | 0.01 | 4.21(1.26,14.03) | 0.02 |

Data are presented as HR (95% CI). Model I adjusted for age, sex and race/ethnicity. Model II adjusted for age, sex, race/ethnicity, education levels, BMI, smoking, HbA1c, TC, hypertension, DM, calcium, phosphorus, phosphorus intake, calcium intake, magnesium intake, CDAI, vitmain D intake, physical activity and poverty income ratio.
